# Design and deposition of a metal-like and admittance-matching metamaterial as an ultra-thin perfect absorber

**DOI:** 10.1038/s41598-017-03392-7

**Published:** 2017-06-08

**Authors:** Yi-Jun Jen, Wei-Chih Liu, Tso-Kuei Chen, Shan-wen Lin, Yi-Ciang Jhang

**Affiliations:** Department of Electro-Optical Engineering, National Taipei University of Technology, No. 1, Sec. 3, Chung-Hsiao E. Rd, Taipei, Taiwan

## Abstract

A stratiform metamaterial, comprising metal and dielectric thin films, exhibits both near-perfect antireflection and strong light extinction to function as a perfect and ultra-thin light absorber. The equivalent admittance and extinction coefficient of the metamaterial are tailored using a visual method that is based on an admittance diagram. A five-layered metamaterial was designed and deposited with a total thickness of 260 nm on a mirror to exhibit strong and wide angle absorption over wavelengths from 400 nm to 2000 nm. A seven-layered metamaterial with a total thickness of less than 200 nm was designed and deposited to have equivalent admittance around unity and an extinction coefficient that is comparable to that of metal. Such a metal-like metamaterial exhibits low reflectivity so couples most visible light energy into the films and dissipates energy with an equivalent skin depth of less than 55 nm over visible wavelengths.

## Introduction

Metals have very high conductivity so their unbounded electrons are driven by illuminating electromagnetic waves, dissipating the energy of those waves within a small depth below their surfaces. However, this strong light dissipation absorbs only a small amount of incident light energy because absorption is accompanied by high reflectivity, which causes metallic surfaces to act as excellent mirrors. Scientists and engineers seek to develop a material that exhibits strong light dissipation but also low reflectivity. A very thin layer of such a material should absorb the energy of incident light in a broadband and over a wide range of angles of incidence. In this work, the equivalent admittance and refractive index of a stratiform metamaterial that comprises alternatively arranged metal and dielectric films are tailored for admittance matching to the admittance of the cover medium. The large imaginary part (extinction coefficient) equivalent refractive index of such a metamaterial causes it to have a skin depth that is close to that of a metal. The designed seven-layered structure with a thickness of only 180 nm was deposited on a glass substrate to absorb over 92% of light at wavelengths from 400 nm to 700 nm and angles of incidence from 0° to 70°.

Highly efficient light absorbers have a variety of crucial applications, such as in thermophotovoltaics^1^, photodetection^2^, thermal imaging^3^ and thermal emission^4^. Scientists and engineers urgently require a broadband and omnidirectional absorber, called a perfect absorber, with a thin and compact structure^5^. An ultra-thin perfect absorber must simultaneously exhibit perfect antireflection and strong light dissipation. However, strong energy dissipation in a homogeneous layer requires a large extinction coefficient, which favors reflection of a significant fraction of incident light, reducing absorption. One decade ago, a nanostructure that mimics the eyes of a moth^6^, with a grade refractive index profile, was developed to exhibit near-perfect antireflection and absorption. However, the grade refractive index requires the thickness of the perfect absorber to be sufficiently high to reduce reflection over a wide range of wavelengths and angles of incidence^7^. Numerous attempts have recently been made to reduce the thickness of the absorber by various mechanisms, including multiple surface plasmon resonance^8^, slow-light effects^9^, and admittance matching^10^. A compact structure that comprises metal and dielectric films, fabricated as a precise optical coating, exhibits strong light absorption^11^. Although several examples of metal-dielectric multilayered absorbers have been proposed^12–14^, a method for developing an ultra-thin layered absorber with respect to a range of wavelength is still lacking.

Extraordinary light absorption is expected from an ultra-thin metamaterial. The permittivity and permeability of a metamaterial must be considered separately^15^. The corresponding optical constants of a metamaterial film, admittance (*E*) and refractive index (*N*), can be derived from permittivity (*ɛ*) and permeability (*μ*) using simple relationships $E=\sqrt{\frac{\varepsilon }{\mu }}$, and $N=\sqrt{\varepsilon \mu }$ ^16^. The admittance equal to that of the incident medium and a strong imaginary part of the refractive index (extinction coefficient) result in simultaneous antireflection and strong light dissipation by a metamaterial layer^17^. The required thickness of the metamaterial layer is then inversely proportional the extinction coefficient. A multilayer that comprises alternatively arranged metal and dielectric films was recently demonstrated as a metamaterial with a negative refractive index over a wide range of angles of incidence^18^. The design and fabrication of optical coatings must be modified to develop various metamaterials. Both admittance and refractive index are included in optical coating design to have a functional metamaterial^19^. Admittance matching of a metamaterial that is composed of repetitions of three-layered symmetrical metal-dielectric-metal structures has been achieved by numerical analysis, but this numerical procedure is approximate and does not yield exact admittance matching^20, 21^. The matching condition must be maintained over a wide range of wavelengths to satisfy the requirement of a perfect light absorber and impedance matching is only one of two important characteristics of an ultra-thin light absorber. A strong equivalent extinction coefficient is also crucial in reducing the total thickness of the absorber.

This work develops a viable method for designing efficient absorbers, based on the normalized admittance diagram^22^. Three-layered, five-layered, and seven-layered symmetrical film stacks are designed for admittance matching to perform highly efficient absorption. The five-layered symmetrical film stack is designed and fabricated for broadband absorption. The seven-layered is designed and fabricated not only for admittance matching but also to have a large equivalent extinction coefficient that is comparable with those of metals. The light dissipation of the layered metamaterial is metal-like but its reflectivity is as low as a highly transparent dielectric medium.

The stratification of dielectric films with a symmetrical elementary cell has been widely utilized in optical filters^23^. Any symmetrical film stack is equivalent to a layer with an equivalent admittance and an equivalent refractive index that are both functions of wavelength as well as the thicknesses and optical constants of the constituent films^24^. For an all-dielectric symmetric film stack, the equivalent admittance and phase thickness are simultaneously real and imaginary for different ranges of wavelength, which are called the pass band and the stop band, respectively^22^. Accordingly, an all-dielectric symmetrical film stack can be used in the design of edge filters. Recently, metal-dielectric stratification has been developed to form a multilayer that is equivalent to a metamateria^25^. Both equivalent admittance and optical phase thickness can be tailored to provide optical properties, such as an equivalent negative index or hyperbolic dispersion.

The product of the characteristic film matrices of the layers of a symmetrical multilayer equals a matrix that has the same form as that of a single layer with an equivalent admittance and optical phase thickness^24^. Equation 1 presents an example of a symmetric three-layered elementary cell, ABA, that is composed of film A and film B.

1 $$[\begin{array}{cc}\cos \,\gamma  & \frac{i}{E}\,\sin \,\gamma \\ iE\,\sin \,\gamma  & \cos \,\gamma \end{array}]=[\begin{array}{cc}\cos \,{\delta }_{A} & \frac{i}{{\eta }_{A}}\,\sin \,{\delta }_{A}\\ i{\eta }_{A}\,\sin \,{\delta }_{A} & \cos \,{\delta }_{A}\end{array}][\begin{array}{cc}\cos \,{\delta }_{B} & \frac{i}{{\eta }_{A}}\,\sin \,{\delta }_{B}\\ i{\eta }_{B}\,\sin \,{\delta }_{B} & \cos \,{\delta }_{B}\end{array}][\begin{array}{cc}\cos \,{\delta }_{A} & \frac{i}{{\eta }_{A}}\,\sin \,{\delta }_{A}\\ i{\eta }_{A}\,\sin \,{\delta }_{A} & \cos \,{\delta }_{A}\end{array}]=[\begin{array}{cc}{M}_{11} & {M}_{12}\\ {M}_{21} & {M}_{22}\end{array}]$$

where γ is the equivalent phase thickness of the stack and *E* is its equivalent admittance. δ and *η* are the phase and admittance of each layer of the stack. The admittance dominates transmission and reflection at the boundaries of the symmetrical film stack and the phase thickness γ dominates wave propagation in the film stack. The refractive index is ${N}_{e}=\frac{\lambda }{2\pi d}\gamma$, where *d* is the total thickness of the film stack.

This work proposes an ultra-thin light absorber by realizing admittance matching and a large extinction coefficient. Ideally, an equivalent metamaterial layer whose equivalent admittance is very close to the admittance of free space, unity, can be designed. When admittance matching is achieved, no reflection occurs at the boundary of the film stack. Admittance matching can be realized by various symmetrical film stacks with different numbers of layers, materials and thicknesses. Admittance matching is favored by making the equivalent extinction coefficient (imaginary part of the equivalent refractive index) as large as possible to reduce the thickness of the film stack.

To achieve broadband high absorption, admittance matching and high extinction of the equivalent layer must be sustained over a wide range of wavelengths. This work develops a design method that is based on the normalized admittance diagram in which the loci of the layered system are traced. The condition for admittance loci that ensures admittance matching is proposed. The extinction coefficients between different admittance matching cases can be qualitatively compared. Broadband admittance matching can be realized by arranging the loci to provide a well compensation effect at wavelengths that deviate from the designed wavelength.

Admittance matching and extinction enhancement are realized using the normalized admittance diagram (NAD). The equivalent admittance under the top surface of a multilayer on a substrate is represented in the NAD. In the building-up process of a multilayer, the variation of the equivalent normalized admittance with the growth of the thin films is calculated and traced on the admittance diagram.

The general formula for tracing the admittance in the NAD is obtained using the transfer matrix method. The tangential components of the magnetic and electric fields at the upper and bottom boundaries of a thin film are related by the characteristic matrix of the thin film^22^, as shown in Eq. 2.

2 $$[\begin{array}{c}{E}_{up}\\ {H}_{up}\end{array}]=[\begin{array}{cc}\cos \,\delta  & (i\,\sin \,\delta )/y\\ iy\,\sin \,\delta  & \cos \,\delta \end{array}][\begin{array}{c}{E}_{bot}\\ {H}_{bot}\end{array}]$$

where *y* is the intrinsic normalized admittance of the film material, defined as the ratio of the tangential component of the refracted magnetic field to that of the refracted electric field at the interface between two half spaces that are occupied by the cover medium and the film material. The admittance of a nonmagnetic material is proportional to the root mean square of the dielectric constant, which is also proportional to the refractive index. In this paper, the admittance was normalized to be the refractive index at normal incidence, the normalized admittance is the same as the refractive index. The *δ* is the phase thickness of the film, which is proportional to the film thickness. The equivalent admittance is defined as the ratio of the tangential component of the magnetic field to that of the electric field at the upper boundary. Accordingly, the equivalent admittance is then a function of *y*, *δ* and the equivalent admittance at the bottom boundary. For a dielectric layer, the admittance *η* = *H*_*up*_/*E*_*up*_ follows a circular loop clockwise in the NAD as the thickness increases. A thin film of an absorbing material with a high extinction coefficient, such as a metal, would have an arc locus that approaches the intrinsic normalized admittance. The locus of a metal film always follows a curve toward the intrinsic normalized admittance of the corresponding bulk metal.

The locus of a dielectric or metallic film that is coated on any substrate can be visually traced in the NAD. The terminal of all loci of constitute films in a multilayer represents the equivalent admittance in the NAD and reveals the optical properties of the multilayer. For example, a multilayer that is composed of high refractive index films and low refractive index films is designed to perform antireflection by having the end of loci of all films to be the admittance of the incident medium to achieve admittance matching^26^. The length of the locus of a thin film is proportional to the phase thickness. The locus of a thin film that is made of a weakly dispersive material would lengthen or shorten as the wavelength of the illuminating light increases or decreases, respectively. Generally, the extended or shortened loci have the terminal of all loci depart from the designated initial point. A low wavelength-dependent reflectance depends on keeping the terminal around the initial point at different wavelengths.

**Figure 1:**
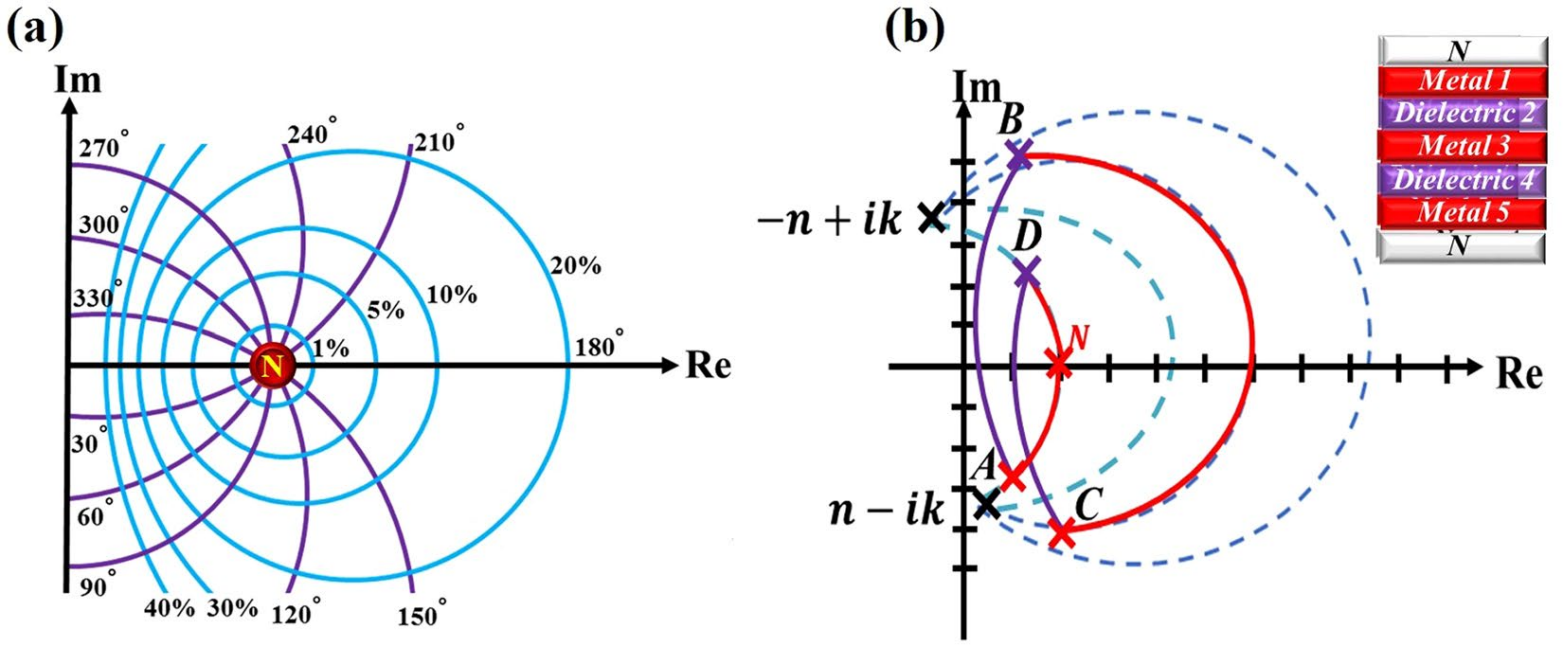
(**a**) Constant reflectance loci (blue lines) and constant phase loci (purple lines), (**b**) loci of an admittance-matching five-layered symmetrical film stack in NAD.

Figure 1 presents the contours of the constant reflectance and the constant phase of the reflection coefficient for an incident medium with refractive index N. The magnitude and phase of the reflection coefficient of a layered system can be derived through visualization rather than calculation. Such a graphical technique is useful in the design of antireflection coatings, high-reflection coatings and other optical filters^22^. Herein, an ultra-thin light absorber that comprises a symmetrical film stack is developed. The idea of admittance matching is based on the fact that, as admittance matching is reached for a metamaterial layer on a substrate whose admittance equals that of the incident medium, the equivalent normalized admittance is the same as that of the incident medium. Here, the metamaterial layer comprises a metal-dielectric symmetric film stack. The admittance loci of the constituent films in an elementary symmetric unit cell must form a closed loop such that the terminal of all loci of the films is the initial point of the admittance (the admittance of the substrate). The locus of a dielectric or metallic film that is coated on any substrate can be visually traced in the NAD. Therefore, in this work, the material of each film is firstly selected such that the terminal of the loci of all films approaches toward the initial point. Next, the thickness of each layer of a symmetrical film stack was tuned to cause the terminal of the loci to be as close as possible to the initial point. Figure 1(b) also schematically shows the loci of a five-layered symmetrical film stack D_5_M_4_D_3_M_2_D_1_, whose equivalent admittance is matched to a particular value of N. The dashed curves in Fig. 1(b) indicate possible loci for a metal film with a complex refractive index n−ik. Secondly, to ensure strong light dissipation, the loci of the metal films should be close to the origin because a small admittance is associated with a strong electric field within the film^27, 28^. Thirdly, broadband admittance matching requires that the terminal of the admittance loci is invariant with wavelength.

**Figure 2:**
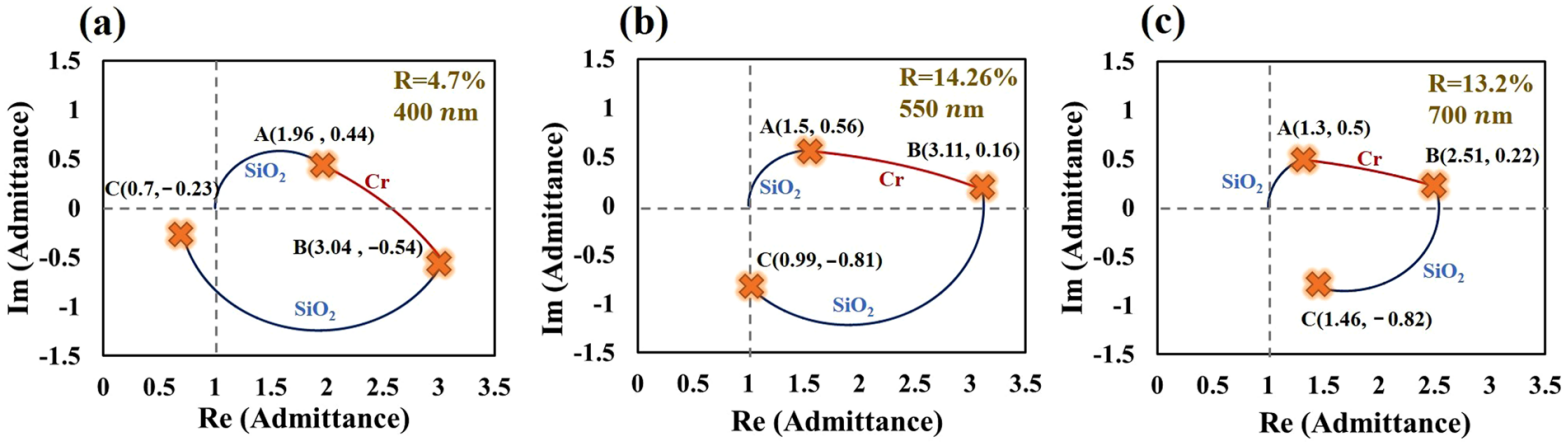
NAD loci of Air/SiO_2_(55 nm)/Cr(6 nm)/SiO_2_(55 nm)/Air system, at wavelengths of 400 nm, 550 nm, and 700 nm, shown as blue (SiO_2_), and red (Cr) curves.

Here, a three-layered symmetric unit cell of the form DMD is presented to approach admittance matching, where M and D are metal and dielectric films, respectively. Admittance matching with air requires that the loci of Air/D_1_M_2_D_3_/Air start at (1, 0) and form a close loop. An example of a system that approaches admittance matching is the Air/SiO_2_(55 nm)/Cr (6 nm)/SiO_2_ (55 nm)/Air system. Figure 2 plots the admittance loci at wavelengths of 400 nm, 550 nm and 700 nm. The locus of the bottom SiO_2_ film follows a circular contour to point A. The locus of the middle layer of Cr follows a spiral contour toward the refractive index of bulk Cr at point B. The top layer of SiO_2_ brings the admittance toward the initial point and the terminal is point C. Notably, the real and imaginary parts of the refractive index of the middle metal must be comparable in magnitude and sufficiently large to ensure that the locus of the metal M_2_ follow a projectile contour that puts the end of the locus of D_3_ near the initial point. At wavelengths of 400 nm, 550 nm and 700 nm, the terminal points are (0.62, −0.25), (0.91, −0.82) and (1.34, −1.08), respectively. Limitations of the materials making achieving perfect admittance matching for a three-layered structure difficult. Therefore, more layers are used in the symmetrical film stack to approach perfect matching over a broad range of wavelengths.

**Figure 3:**
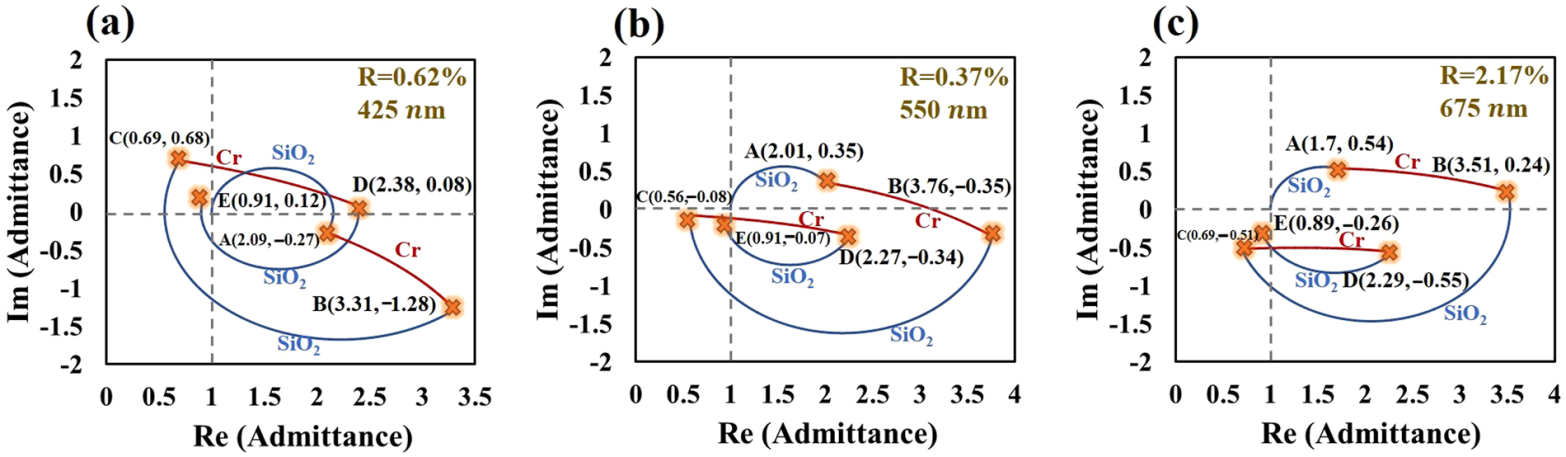
NAD loci of Air/SiO_2_(80 nm)/Cr(6 nm)/SiO_2_(88 nm)/Cr(6 nm)/SiO_2_(80 nm)/Air system at wavelengths of 425 nm, 550 nm, and 675 nm, shown as blue (SiO_2_) and red (Cr) curves.

Based on the above discussion, a five-layered symmetrical film stack is expected to provide more flexibility than a three-layered structure in the design of a broadband absorber. The five-layered symmetrical film stack of SiO_2_(80 nm)/Cr(6 nm)/SiO_2_(88 nm)/Cr(6 nm)/SiO_2_(80 nm) is designed and fabricated to achieve admittance matching. The SiO_2_ and Cr films are deposited using sputtering evaporation. The refractive indices of SiO_2_ and Cr, measured for single layer that was deposited on BK7 glass substrate (See supplementary information), are adopted in the design and simulation. A normalized admittance diagram for five-layered admittance-matching is developed. Figure 3 plots the admittance loci of the Air/D_5_M_4_D_3_M_2_D_1_/Air = Air/SiO_2_(80 nm)/Cr(6 nm)/SiO_2_(88 nm)/Cr(6 nm)/SiO_2_(80 nm)/Air system at wavelengths of 425 nm,550 nm, and 675 nm. At a wavelength of 550 nm, the loci of D_1_ and M_2_ in Fig. 3(b) are similar to those in Fig. 2 but have different lengths. The locus D_3_ brings the admittance to the point C(0.56, −0.08). The locus of M_4_ follows the spiral arc toward the refractive index of Cr and stops at point D(2.27, −0.34). The layer D_5_ forms a circular arc, ending at E(0.91, −0.07), which is close to the initial point. The equivalent admittance, calculated using Eq. 1, is *E* and near-perfect admittance matching is achieved. Notably, locus D_5_ intersects the locus of M_4_, forming a knot, which is critical in extending the range of wavelengths of high absorptance. As the illuminating wavelength decreases or increases, the extended (shortened) loci expand (shrink) the knot but keep the end of all loci remains close to the same original point. The knot acts as a buffer that reduces the variation of equivalent admittance. The Fig. 3(a) and (c) plot the admittance loci at wavelengths of 425 nm and 675 nm.

**Figure 4:**
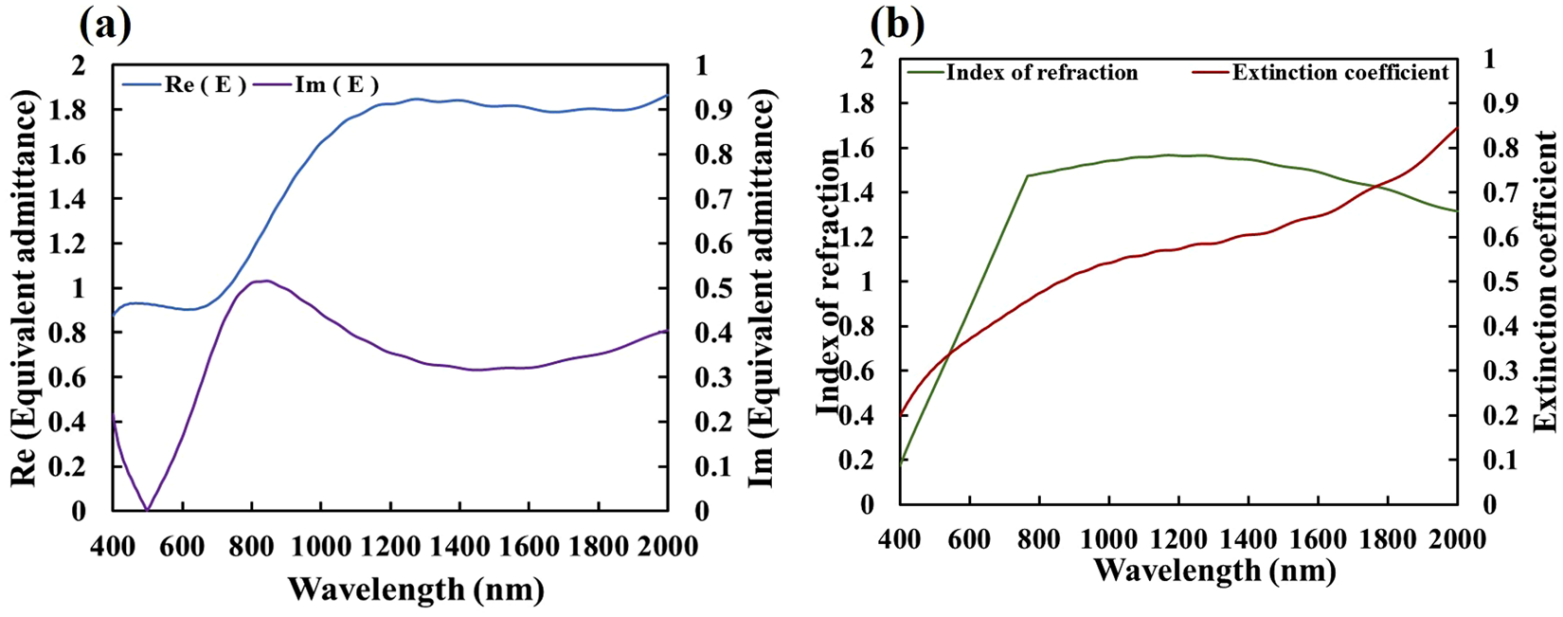
(**a**) Equivalent admittance and (**b**) refractive index of the five-layered metamaterial versus wavelength.

In Fig. 3, the locus of M_4_ is closer to the origin than is that of M_2_, so the electric field intensity in M_4_ is stronger than that in M_2_. The visualized proposed design method can be used not only for admittance matching but also to compare light extinction between two cases of matching. The equivalent admittance (*E*) and extinction coefficient (${N^{\prime\prime} }_{e}$) of the unit-cell metamaterial are calculated and shown in Fig. 4. The real part of the equivalent admittance varies within 0.9 ± 0.03 over the range of visible wavelengths. The imaginary part of the equivalent admittance is less than 0.38 in the visible regime and less than 0.2 at wavelengths from 403 nm to 615 nm. At wavelengths longer than 1000 nm, the equivalent admittance does not match unity. The real part of the equivalent admittance varies within 1.28 ± 0.07 over wavelengths from 1230 nm to 2000 nm. The extinction coefficient increases from 0.198 at a wavelength of 400 nm to 0.845 at a wavelength of 2000 nm. The index of refraction increases from 0.171 at a wavelength of 400 nm to 1.44 at a wavelength of 757 nm and remains within the range 1.44 ± 0.12 from 757 nm to 2000 nm.

**Figure 5:**
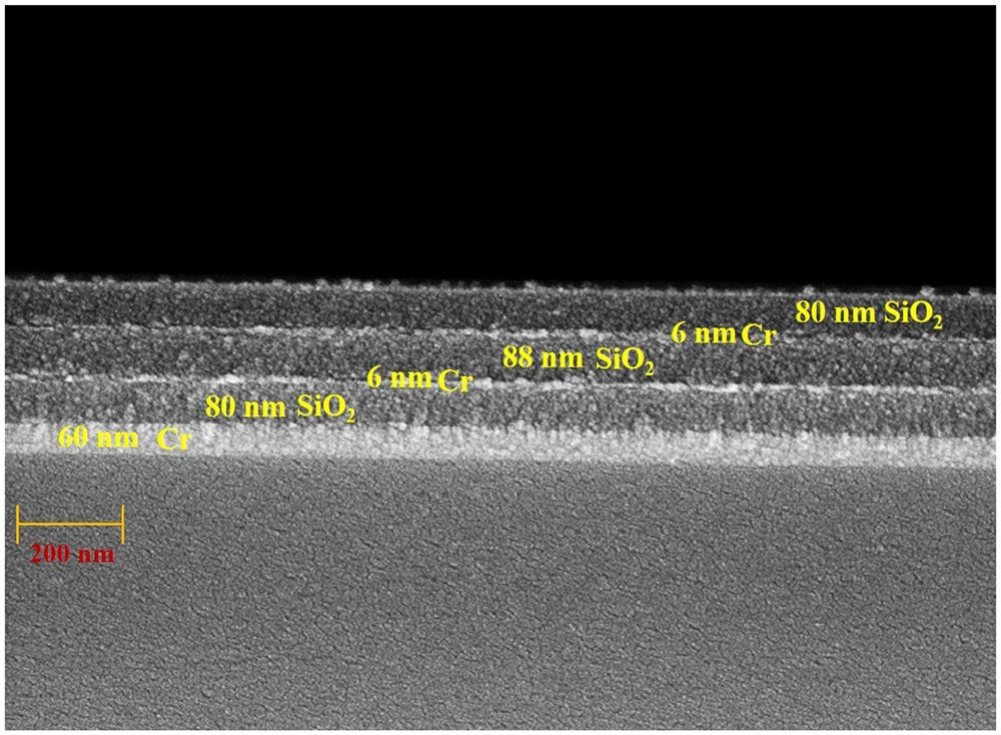
Scanning electron microscope (SEM) image of the five-layered symmetrical multilayer on a 60 nm Cr layer.

**Figure 6:**
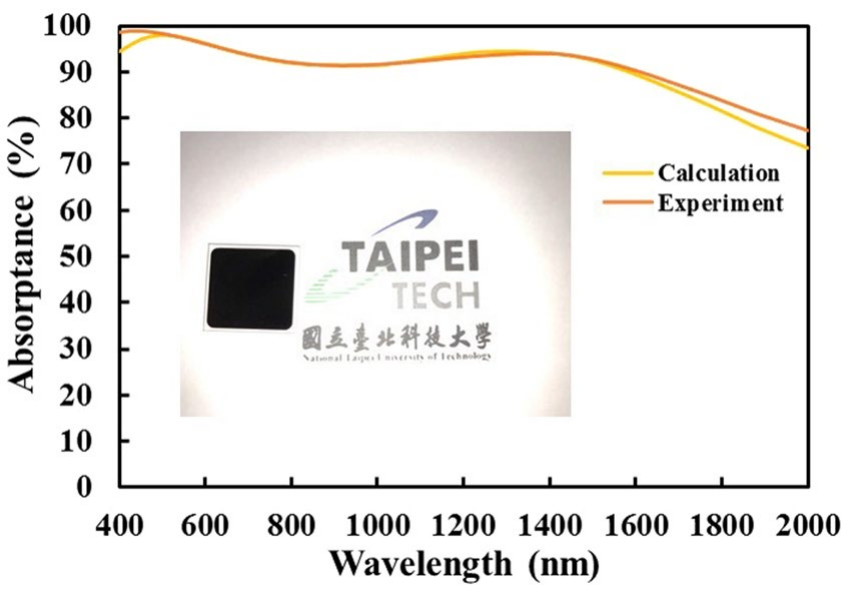
Measured and calculated absorptance spectra of Air/SiO_2_(80 nm)/Cr(6 nm)/SiO_2_(88 nm)/Cr(6 nm)/SiO_2_(80 nm)/Cr(60 nm) glass system at normal incidence. Inset shows a photograph of fabricated absorber device.

Arranging several units of the five-layered structure on a transparent glass substrate reduces the transmission and yields weak reflection. Five periods of the unit cell on a glass substrate reduce the transmittance at normal incident to less than 0.066% on average and increases the average absorptance to 99% over the visible wavelengths. (See Supplementary information.) The total thickness of the five-unit multilayer is 1300 nm. To fabricate a light absorber that is as thin as possible, the symmetrical film stack can be arranged on a highly reflective coating to minimize required unit cells. An ultra-thin absorber that comprises one of the aforementioned unit cell and a coated reflective mirror in the form of a Cr film is deposited using sputtering evaporation as Air/SiO_2_(80 nm)/Cr(6 nm)/SiO_2_(88 nm)/Cr(6 nm)/SiO_2_(80 nm)/Cr(60 nm)/Bk7 glass. Figure 5 shows the cross section of scanning electron microscopy image. The thickness of the five-layered stack is 260 nm. Figure 6 shows the calculated and measured absorptance spectra of the five layers on the Cr mirror at normal incidence. The average absorptance exceeds 91.23% over all wavelengths. The absorptance exceeds 90% over wavelengths from 400 nm to 1615 nm. The measured and calculated results are highly in agreement. According to the analysis of the thickness tolerance for the five-layer system, a 10% error in the thicknesses of the deposited middle SiO_2_, non-middle SiO_2_ and Cr layers would cause discrepancies in the average absorptance between measured and simulated spectra at normal incidence, of approximately 0.72%, 1.1% and 0.24%, respectively. Although the admittance did not match to that of air at wavelengths from 1000 to 2000 nm, the reflection was still low and high absorption was achieved, because destructive interference in this range of wavelengths. At a wavelength of 1300 nm, the phase difference between the first two ordered reflected waves was 173.60°. Figure S2 in the supplementary information presents the destructive interference phenomena at wavelengths of 1100 nm, 1200 nm, 1300 nm and 1400 nm.

**Figure 7:**
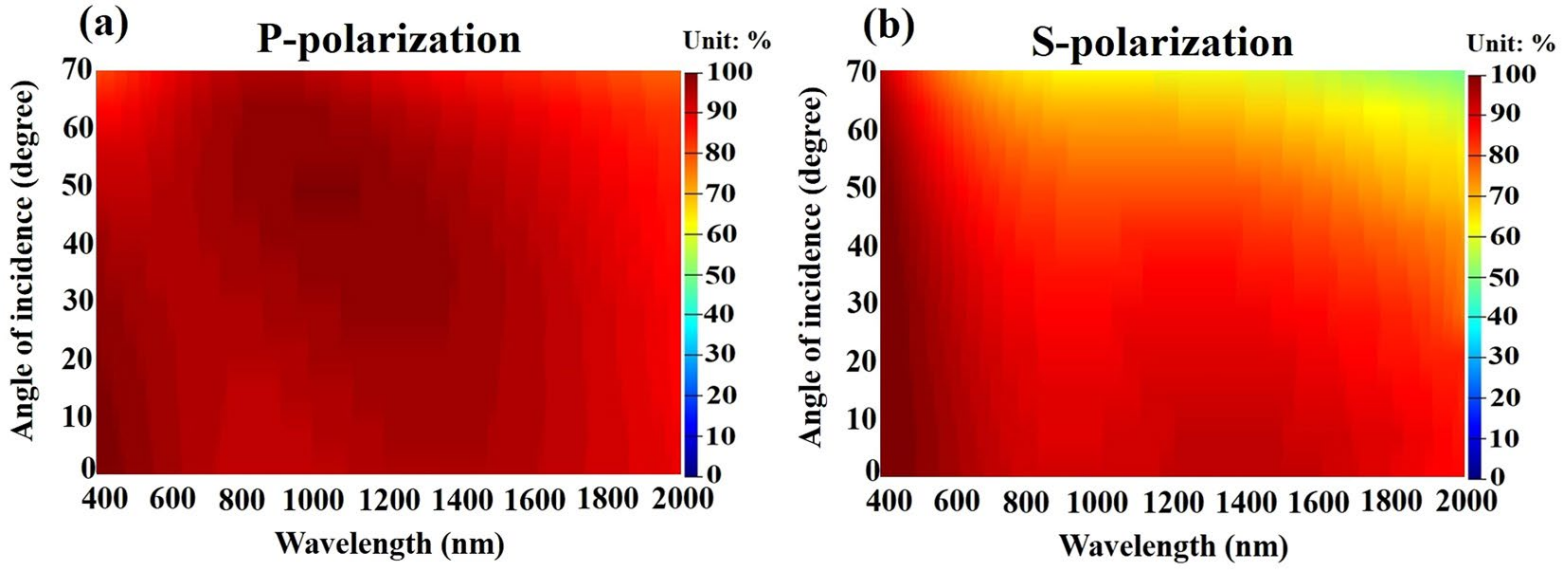
Experimental absorptance as functions of wavelength and angle of incidence for Air/SiO_2_(80 nm)/Cr(6 nm)/SiO_2_(88 nm)/Cr(6 nm)/SiO_2_(80 nm)/Cr(60 nm) glass system; (**a**) p-polarized absorptance and (**b**) s-polarized absorptance.

Figure 7 plots the measured absorptance for wavelengths from 400 nm to 2000 nm and angles of incidence from 20° to 70°. As expected, polarized light is strongly absorbed at visible wavelengths over a wide range of angles of incidence from 20° to 60°. The p-polarized absorption is strong at all such wavelengths and angles the average p-polarized absorptance is 93.3%. The s-polarized absorption is weaker than p-polarized absorption and the absorption decays with the angle of incidence. At angles of incidence between 45° and 70°, the s-polarized absorption decays with wavelength. However, the average absorptance of both polarization states is 88.93% over all visible wavelengths and specified angles of incidence. In many instances of antireflection, the p-polarized reflectance is lower than the s-polarized reflectance at large angles of incidence because at any interface, the p-polarized reflectance is reduced to a minimum at the Brewster angle whereas s-polarized reflectance increases with the angle of incidence. Therefore, the fact that narrower range of angle of incidence of s-polarized absorptance spectrum than that of p-polarized absorptance spectrum comes from the increasing in s-polarized reflectance.

**Figure 8:**
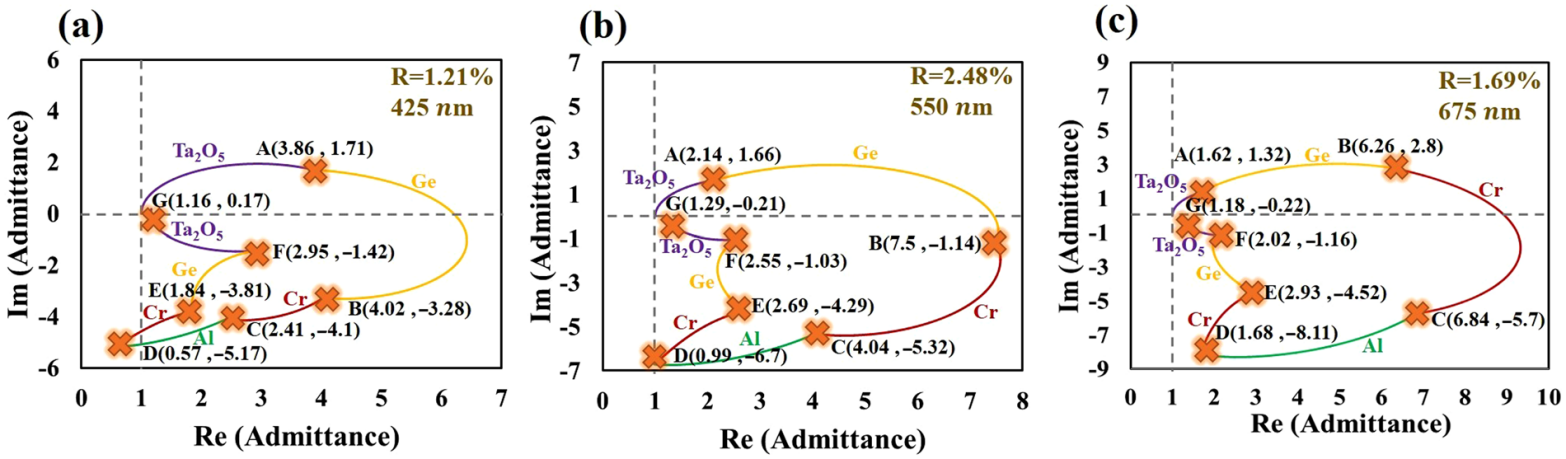
Normalized admittance diagram of Air/Ta_2_O_5_(40 nm)/Ge(20 nm)/Cr(15 nm)/Al(30 nm)/Cr(15 nm)/Ge(20 nm)/Ta_2_O_5_(40 nm)/Air system at 425 nm, 550 nm, and 675 nm, shown in purple (Ta_2_O_5_), yellow (Ge), red (Cr), and green(Al) lines.

A seven-layered symmetrical film stack herein is found to exhibit a high extinction coefficient as a bulk metal at visible wavelengths. The seven-layered symmetrical film stack is fabricated by sputtering evaporation as Ta_2_O_5_(40 nm)/Ge(20 nm)/Cr(15 nm)/Al(30 nm)/Cr(15 nm)/Ge(20 nm)/Ta_2_O_5_(40 nm). The refractive index of each film as a single layer that is deposited on a glass substrate is measured using an ellipsometer to simulate the admittance loci and absorptance of the symmetrical film stack(See supplementary information.). A normalized admittance diagram for seven-layered admittance-matching is developed. Figure 8 plots the admittance loci of the Air/D_7_M_6_M_5_M_4_M_3_M_2_D_1_/Air = Air/Ta_2_O_5_(40 nm)/Ge(20 nm)/Cr(15 nm)/Al(30 nm)/Cr(15 nm)/Ge(20 nm)/Ta_2_O_5_(40 nm)/Air system at wavelengths of 425 nm, 550 nm, and 675 nm. At a wavelength of 550 nm, the layers of M_2_ and M_3_ form a relatively large contour that intersects the real axis at the point (7.45, 0) and ends at point C(4.04, −5.32). The loci of M_4_, M_5_, and M_6_ at positions that correspond to low magnitudes of admittance, indicating efficient light dissipation. The top layer of D_6_ brings the terminal of all loci to point G(1.29, −0.21), which is near the initial point. At wavelengths of 425 nm and 675 nm, the loops that are composed of all of the loci that are shown in Fig. 8(a) and (c) are expanded and shrink, respectively, keeping the terminal around the initial point. In this case, all loci form an almost closed loop. The extended (shortened) loci expand (shrink) the loop but the end of the loci remains close to the same original point. Since the index of refraction of Al increases from 0.55 at a wavelength of 425 nm to 1.6 at a wavelength of 675 nm, the phase thickness decreases as the wavelength increases. Therefore, the locus of the Al film shortens as the wavelength of the illuminated light decreases. The Al film with a different trend of locus variation from other films acts as a buffer layer that reduces the variation of the terminal point. On the other hand, as the wavelength decreases, the loci of the top two layers extend to the inside of the loop instead of pushing the terminal apart from the initial point. Therefore, the design shown in admittance diagram presents a well compensation effect to achieve broadband low reflectivity.

**Figure 9:**
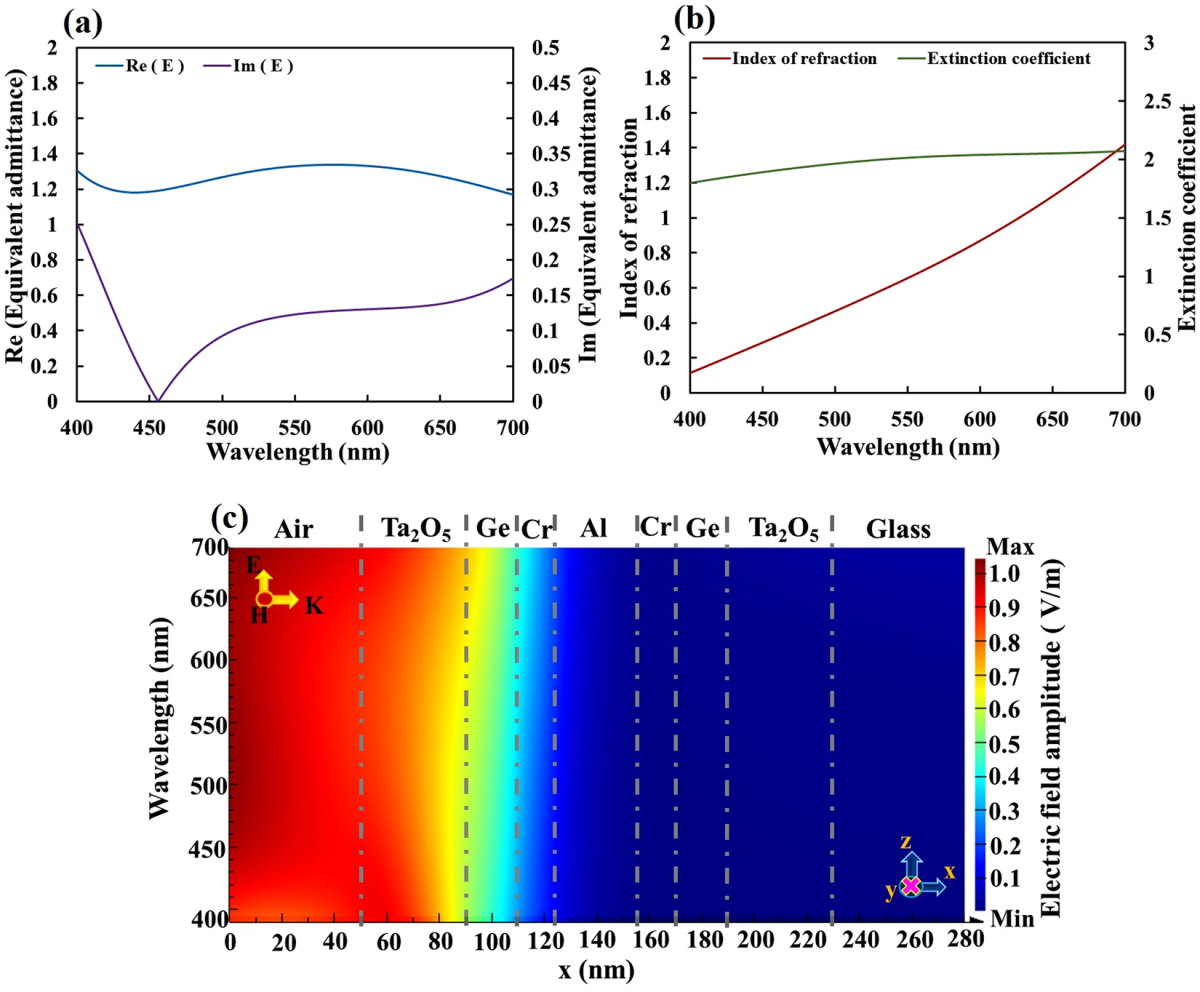
(**a**) Equivalent admittance, (**b**) equivalent refractive index, and (**c**) Electric field amplitude as function of wavelength.

The equivalent admittance (*E*) and extinction coefficient (${N}_{e}^{^{\prime\prime} }$) of the unit-cell metamaterial are calculated using Eq. (1) as functions of wavelength, as shown in Fig. 9. The proposed designed multilayer stack exhibits near-perfect admittance matching, and has a high extinction coefficient, which is characteristic of metal. The real part of equivalent admittance varies within 1.25 ± 0.08 over the whole range of visible wavelengths. The imaginary part of the equivalent admittance is less than 0.25 over the range of visible wavelengths. At wavelengths from 430 nm to 500 nm, Im (*E*) is less than 0.1. The index of refraction linearly from 0.11 at a wavelength of 400 nm to 1.42 at a wavelength of 700 nm. The extinction coefficient increases from 1.8 at a wavelength of 400 nm to 2.0 at a wavelength of 530 nm, and remains around 2.1 at wavelengths from 500 nm to 700 nm. The high extinction coefficient around 2.1 at a wavelength of 700 nm yields a skin depth of 53 nm. The skin depth is also obtained by near-field simulation. The skin depths at wavelengths of 400 nm and 550 nm are 35 nm and 43 nm, respectively. Figure 9(c) plots the distribution of the field amplitude in the multilayer that is illuminated by electromagnetic waves with an electric field amplitude of unity.

**Figure 10:**
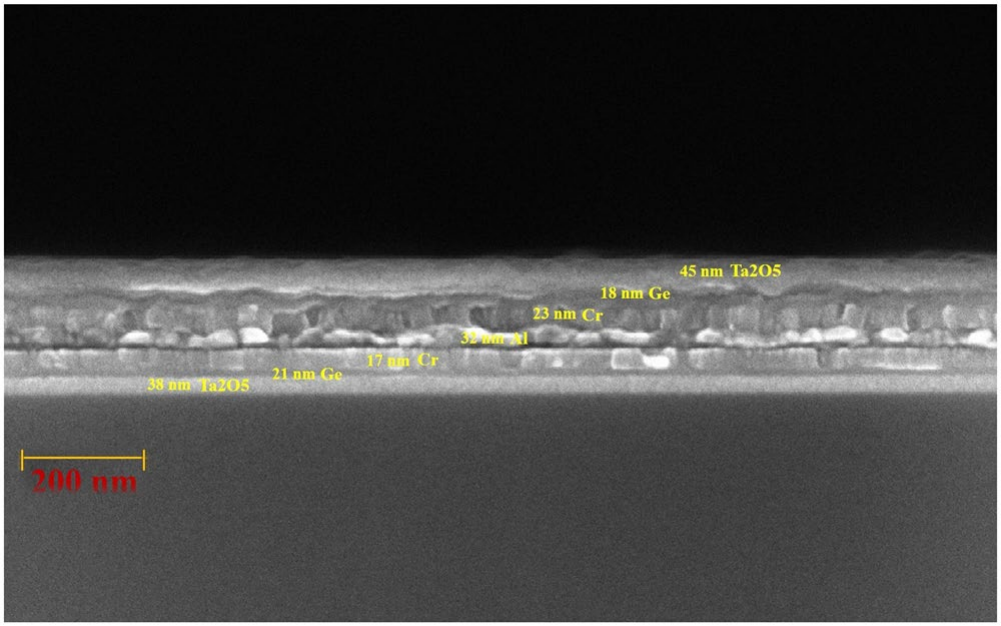
The cross-sectional SEM image of the seven-layered structure.

**Figure 11:**
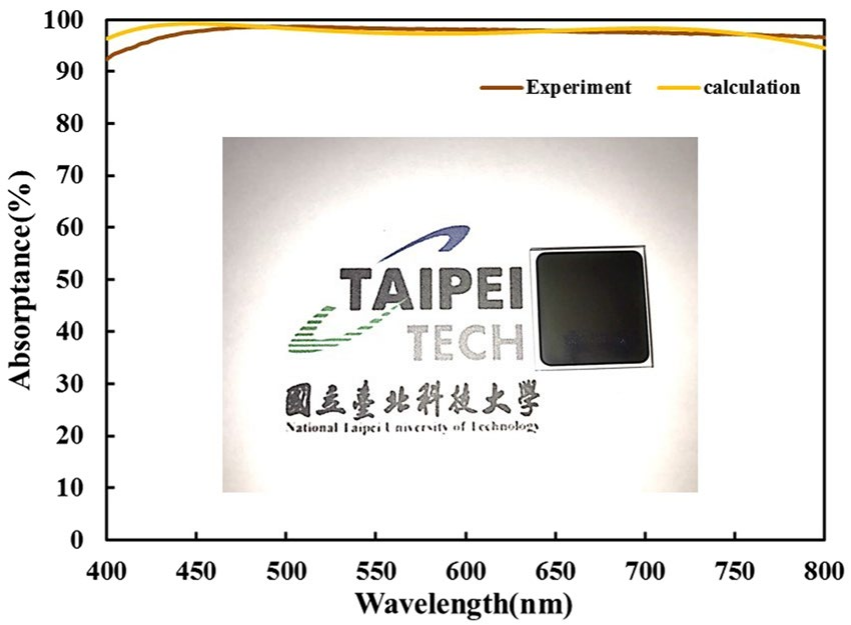
Measured and calculated absorptance spectra of Air/Ta_2_O_5_(40 nm)/Ge(20 nm)/Cr(15 nm)/Al(30 nm)/Cr(15 nm)/Ge(20 nm)/Ta_2_O_5_(40 nm)/glass at normal incidence. Inset shows a photograph of the fabricated absorber device.

**Figure 12:**
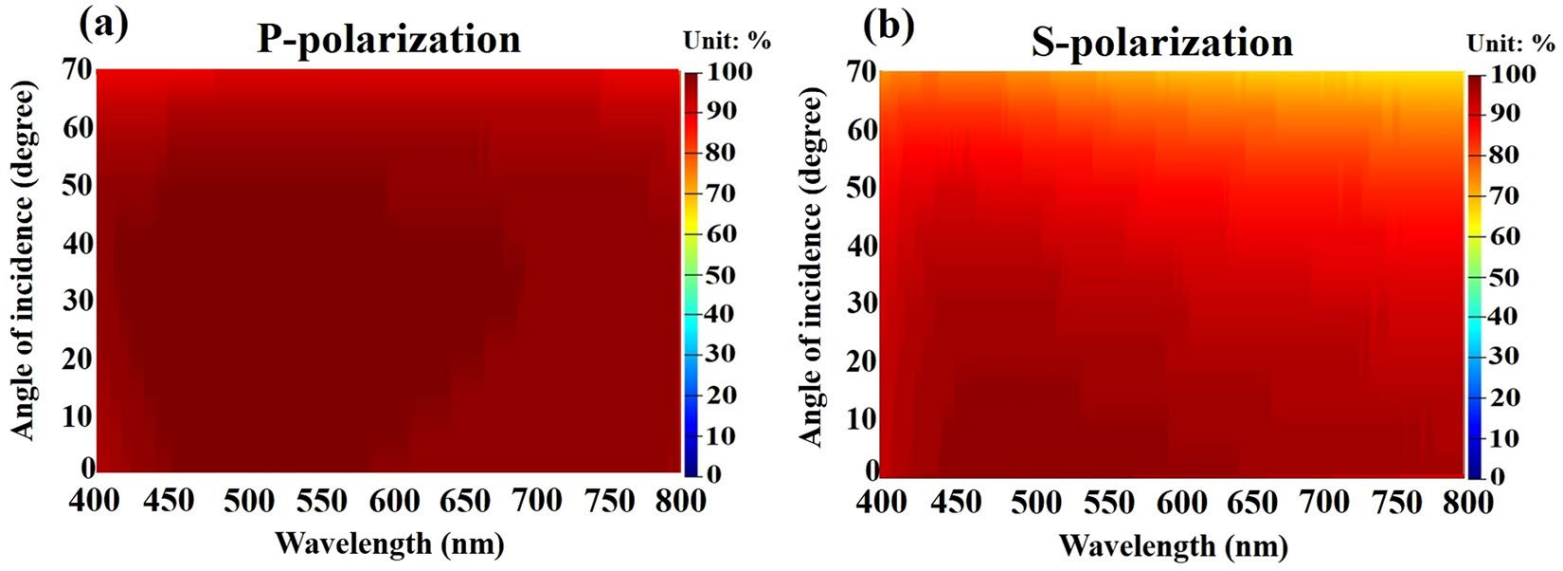
Measured (**a**) p-polarized and (**b**) s-polarized absorptance of seven-layered system as functions of wavelength and angle of incidence.

The designed seven-layered metamaterial was deposited on a transparent BK7 glass substrate by sputtering evaporation. Figure 10 shows the cross section of scanning electron microscopy image. The thickness of the seven-layered stack is 194 nm. Figure 11 shows the measured and calculated absorptance spectra at normal incidence. The figure indicates that high absorption is achieved without a mirror underneath the metamaterial. The measured absorptance exceeds 92% over the entire range of visible wavelengths and the absorptance exceeds 98% at wavelengths from 456 nm to 643 nm. The discrepancy between measured and calculated absorptance spectra arises from the error in the thickness of the Ta_2_O_5_ layer. According to the analysis of the thickness tolerance for seven-layer absorber, a 10% error in the thicknesses of the deposited Ta_2_O_5_, Ge and Cr layers would cause discrepancies in the average absorptance between measured and simulated spectra at normal incidence, of approximately 1.54%, 0.35% and 0.11%, respectively. Figure 12 plots the measured absorptance for wavelengths from 400 nm to 700 nm and angles of incidence from 20° to 70°. The sever-layered stack exhibits excellent angular tolerance and polarization-independent performance. The absorption of p-polarized waves exceeds 92% for wavelengths from 400 nm to 800 nm even at an angle of incidence of 65°. The absorption of s-polarized waves exceeds 95% for wavelengths from 427 nm to 572 nm and angles of incidence from 20° to 40°. The s-polarized absorption is slightly weaker than the p-polarized absorption at short wavelengths around 400 nm and long wavelengths around 800 nm.

In conclusion, a broadband antireflection equivalent layer, comprising a symmetric film stack, is developed in the admittance diagram. The condition for admittance matching in the diagram is elucidated to facilitate the design of an admittance matching layer with any number of thin films to form a symmetric unit cell. The equivalent layer exhibits not only near-perfect antireflection but also strong light extinction. The combination of these optical properties, which traditionally conflict with each other, enables an ultra-thin light absorber to be fabricated. An example demonstrates that a five-layered metamaterial provides more flexibility than a three-layered metamaterial in the design of a broadband and wide-angle admittance matching layer. Admittance matching at visible wavelengths and destructive interference at infrared wavelengths provide high absorption over a broad band. A seven-layered metamaterial is designed with a large extinction coefficient and admittance matching with air. With a thickness of only 192 nm, the metal-like metamaterial absorbs more than 97% of the energy of incident visible light. Based on this work, more thin films with a tailored refractive index and equivalent admittance can be designed, opening up a new avenue in the development of optical coatings for novel optical devices.

## Electronic supplementary material

Design and deposition of a metal-like and admittance-matching metamaterial as an ultra-thin perfect absorber
