# Supplementary material for: Design and deposition of a metal-like and admittance-matching metamaterial as an ultra-thin perfect absorber
Source: Sci Rep. 2017 Jun 8;7:3076. doi: 10.1038/s41598-017-03392-7 (PMC5465059; doi:10.1038/s41598-017-03392-7)
Supplement: Supplementary file 1 — Design and deposition of a metal-like and admittance-matching metamaterial as an ultra-thin perfect absorber [file 41598_2017_3392_MOESM1_ESM.pdf]

Supplementary Information:

Design and deposition of a metal-like and admittance-matching metamaterial as an ultra-thin perfect absorber

Yi-Jun Jen[1*], Wei-Chih Liu[1], Tso-Kuei Chen[1], Shan-wen Lin[1] and Yi-Ciang Jhang[1]

[1]Department of Electro-Optical Engineering, National Taipei University of Technology, No. 1, Sec. 3, Chung-Hsiao E. Rd. Taipei, Taiwan (106)

*Corresponding Email: jyjun@ntut.edu.tw

S1. Refractive indices of thin SiO_2(80 nm), Cr(6 nm), Ta_2O_5(40 nm), Cr(15 nm), Ge(20 nm), and, Al(30 nm) films are shown in Figure S1. The refractive index of each film was measured for a single layer that was deposited on a BK7 glass substrate. We employed a sputtering system (ULVAC Inc.) to deposit each film by tuning the power applied to the target and deposition time to control the thickness. The refractive index of each film was measured using a J. A. Woollam M2000 spectroscopic ellipsometer and a Jobin Yvon iHR320 spectrophotometer.

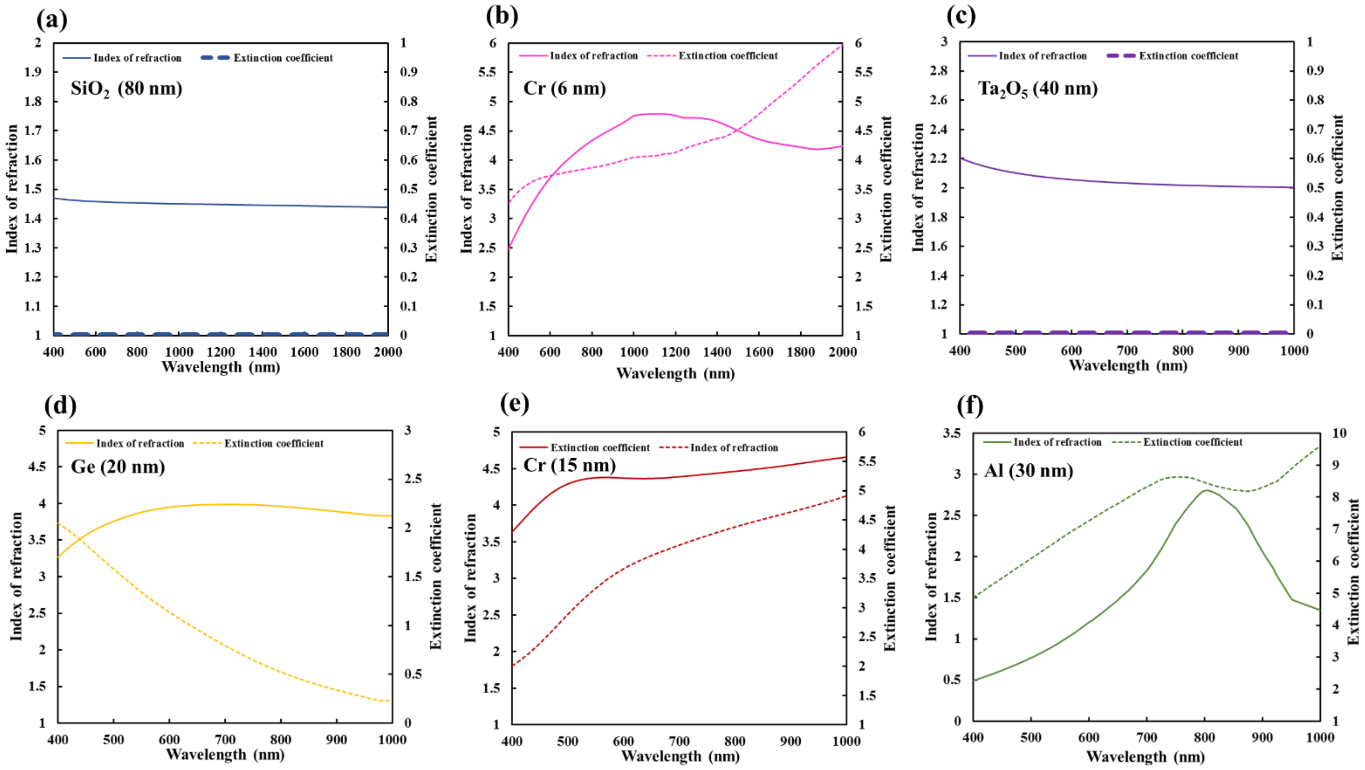

Figure S1 Measured refractive indices as functions of wavelength.

S2. Absorptance spectra of one period, two periods and five periods of the unit cell on a BK7 glass substrate.

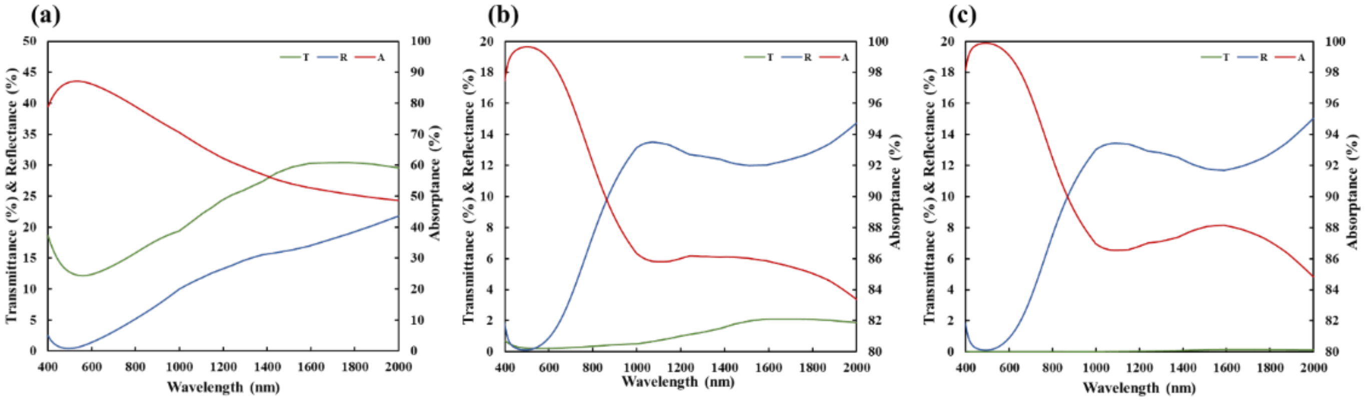

Figure S2. Transmittance(T), reflectance(R) and absorptance(A) spectra of Air/[SiO_2(80 nm)/Cr(6 nm)/SiO_2(88 nm)/Cr(6nm)/SiO_2(80 nm)]^x/BK 7 glass system; (a) one period (x=1), (b)three periods (x=3), and (c)five periods (x=5)

S3. Wave tracing through five-layered structure on Cr mirror at wavelengths of 1100 nm, 1200 nm, 1300 nm, and 1400 nm

From the retrieved values of the equivalent refractive index and admittance, the low reflectivity at infrared regime can be understood by tracing the propagation of light through the multilayer. For example, as shown in Figure S3, when the multilayer is normally illuminated by light with an electric field amplitude of unity and a wavelength of 1300 nm, the light penetrates the top interface of the multilayer with transmission coefficient $0.81\angle 19.43°$. The first-order reflected wave has an amplitude $r_a = 0.36\angle 131.09°$. From the top interface to the bottom interface, the wave propagates with a phase change of $111.36°$ and the field amplitude decays to $0.38\angle -91.93°$. The reflection coefficient and transmission coefficient at the bottom interface are $0.61\angle 168.64°$ and $0.42\angle 16.76°$, respectively. The electric field of the transmitted wave has an amplitude $0.16\angle -75.14°$. The reflected wave from the bottom interface propagates backward with an initial field amplitude of $0.24\angle 79.73°$. When the reflected wave returns to the top interface, the transmitted wave is the second-order reflected wave with an amplitude $r_{aa} = 0.14\angle -42.51°$, and interferes destructively with the first-order reflected wave. The reflected wave from the top interface has an initial magnitude of $0.03\angle -68.88°$, decaying to $0.02\angle 179.65°$ when the wave again reaches the bottom interface. The first-order transmitted wave contributes most to the transmittance.

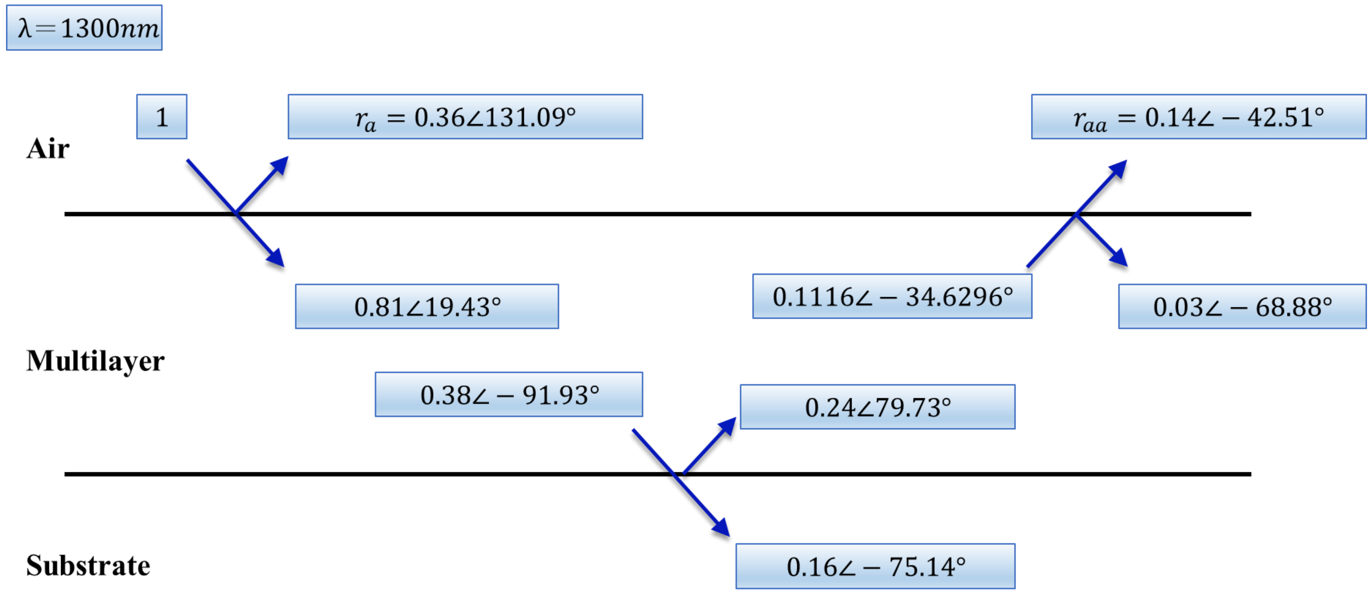

Figure S3. Wave tracing through Air/SiO_2(80 nm)/Cr(6 nm)/SiO_2(88 nm)/Cr(6 nm)/SiO_2(80 nm)/Cr(60 nm)glass system that is normally illuminated by light with an electric field amplitude of unity at a wavelength of 1300 nm

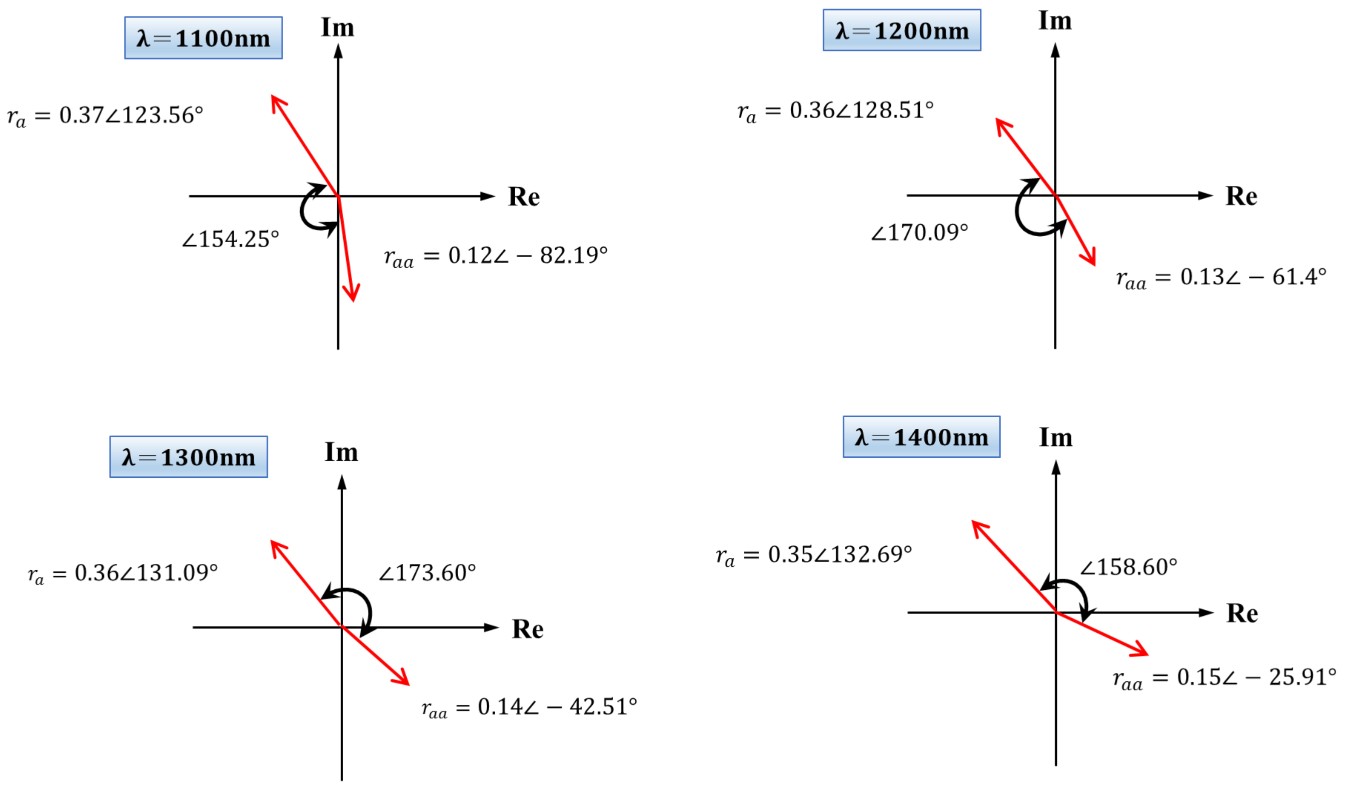

Figure S4. Destructive interference of first-order reflected wave of amplitude r_a and second-order reflected wave of amplitude r_{aa} in complex plane**.**
